# Clinical characteristics of infants with port-wine stain and glaucoma secondary to Sturge–Weber Syndrome

**DOI:** 10.1186/s12886-022-02476-x

**Published:** 2022-06-09

**Authors:** Honggai Yan, Man Hu, Yanhui Cui, Li Li, Tianwei Liang

**Affiliations:** Department of Ophthalmology, Beijing Children’s Hospital, Capital Medical University, National Center for Children’s Health, Beijing, 100045 China

**Keywords:** Sturge–Weber Syndrome, Port-wine stain, Glaucoma

## Abstract

**Background:**

Sturge–Weber Syndrome (SWS) is a rare disease involving the eye, skin, and brain. Port-wine stain (PWS) and glaucoma are common clinical manifestations. This study analysed the clinical characteristics of infants with PWS and glaucoma secondary to SWS.

**Methods:**

Children with PWS and glaucoma secondary to SWS were enrolled. Data were extracted from ophthalmic and systemic examination findings. Ocular examinations included intraocular pressure, anterior segment and fundus examination, and ocular A-scan and B-scan ultrasonography.

**Results:**

Fifty-seven patients were included, with a mean age of 9.9 ± 11.9 months, and 34 (59.6%) patients were male. In all, 61 eyes were diagnosed with glaucoma. Forty-one patients (71.9%) had unilateral facial PWS and glaucoma occurred on the same side. Eight patients (14.0%) had Mongolian spots and ten patients (17.5%) had epilepsy. Corneal changes included corneal oedema (*n* = 36 eyes, 59.0%), corneal opacity (*n* = 15 eyes, 24.6%), and Haab lines (*n* = 13 eyes, 21.3%). Mean corneal diameter and thickness in the eyes with glaucoma was larger than those in the unaffected eyes (12.2 ± 0.7 mm vs 10.8 ± 0.6 mm, *P* < 0.001; 681.2 ± 106.4 µm vs 578.2 ± 58.2 µm, *P* < 0.001). The eyes with glaucoma had higher IOP and larger axial length and C/D ratio (19.3 ± 6.2 mmHg vs 11.6 ± 4.2 mmHg, *P* < 0.001; 21.23 ± 1.93 mm vs 19.68 ± 1.61 mm, *P* < 0.001; and 0.57 ± 0.18 vs 0.24 ± 0.15, *P* < 0.001). Thirty-three (57.9%) and 25 (43.9%) patients showed diffuse choroidal haemangioma (DCH) and conjunctival/episcleral haemangiomas, respectively. Ten patients (17.5%) showed iris anterior insertion or hyperpigmentation in the anterior chamber angles. Six of them had Mongolian spots at the same time.

**Conclusions:**

Monocular glaucoma, DCH, and conjunctival/episcleral haemangiomas are common in SWS patients with PWS and glaucoma. Glaucomatous eyes have larger corneal diameter and axial length and thicker cornea. Patients with Mongolian spots have higher incidence of iris anterior insertion or hyperpigmentation in anterior chamber angle.

## Background

Sturge–Weber syndrome (SWS) is a rare disease that is classically associated with the skin, eyes, and brain. The most common characteristics are glaucoma, diffuse choroidal haemangiomas (DCH), occipital leptomeningeal angioma, and vascular malformations of the facial skin called port-wine stain (PWS) that involve the trigeminal nerve distribution area [1]. The incidence of SWS is between 1:20,000 and 1:50,000 [1]. SWS is divided into three types: type 1 includes leptomeningeal and facial angioma with or without glaucoma, which is the most common; type 2 presents with facial angioma as the most prominent manifestation with or without glaucoma, but no brain involvement; and type 3 only includes leptomeningeal angioma [2, 3]. Some genetic mutations were identified in this syndrome, but no specific inheritance pattern has yet been found [4].

Ocular involvement is observed in a significant portion of patients with SWS. The most commonly reported ocular manifestation is glaucoma. The incidence of glaucoma is among 30–70% [5, 6]. DCH is a benign vascular tumour from choroidal vasculature. The prevalence is 40–50% in patients with SWS and is usually on the ipsilateral side with PWS [6]. Other ocular manifestations such as conjunctival or episcleral haemangiomas, retinal vascular tortuosity, iris heterochromia, cataract, and strabismus have also been occasionally reported [5, 7–9].

Although the ocular and systemic manifestations of patients with SWS have been reported in detail and are well recognised, the clinical features of SWS patients with secondary glaucoma in infancy are unique, and related reports are rare. Thus, the purpose of this study was to analyse the clinical characteristics of infants with PWS and glaucoma secondary to SWS.

## Methods

Children with PWS and glaucoma secondary to SWS treated at Beijing Children’s Hospital from 2015 to 2019 were enrolled in the study. The study was conducted in adherence with the tenets of the Helsinki Declaration, and consent was obtained from the parents or legal guardians of all patients. The inclusion criteria were as follows: (1) PWS on facial skin; (2) diagnosis with glaucoma; and (3) age ≤ 4 years. The exclusion criteria were as follows: (1) family history of glaucoma, (2) history of ocular surgery; and (3) ocular trauma. Data extracted from the reviewed records on the first visit, ophthalmic examination, and systemic examination findings. For all patients, age, sex, family history, and systemic and ocular characteristics were recorded. All ophthalmologic examinations were performed under oral sedation and included the following: anterior segment measurement by a portable slit lamp microscope (YZ3; 66 Vision-Tech, Co. Ltd., Suzhou, China); fundus and anterior chamber angle examinations using a retcam (RetCam 3; Clarity Medical Systems, Co. Ltd., USA) or an indirect ophthalmoscope; IOP measurement by Icare rebound tonometer (Icare Finland, Oy); ocular axial length by A-scan ultrasonography (AL-100, TOMEY Co. Ltd., Japan); corneal diameter by ruler; and evaluation of choroidal haemangioma by ocular B-scan ultrasonography (Eye cubed, Ellex Mesical Pty, Italy).

Glaucoma was diagnosed if at least two of the following criteria of the Childhood Glaucoma Research Network (CGRN) [10] were met: (i) IOP on the corresponding side of PWS was > 21 mmHg or if the IOP on the ipsilateral side of PWS was higher than the contralateral side by > 6 mmHg; (ii) corneal diameter enlargement (neonate: > 11 mm, 1-year-old child: > 12 mm, any age: > 13 mm); and (iii) the cup-to-disc ratio (C/D) was > 0.6 or the asymmetry was > 0.2 between the affected eye and contralateral eye.

Statistical analysis was performed using the SPSS software (version 16.0; IBM Corporation, Armonk, NY, USA). Frequency (n%) and descriptive statistics were used for data analysis. Demographic and clinical characteristics including age, IOP, corneal diameter, corneal thickness, axial length, and C/D ratio were compared using independent-sample *t*-tests; the results were presented as the mean ± standard deviation. A *p*-value of < 0.05 was considered to indicate statistical significance.

## Results

Fifty-seven paediatric patients (59.6% male, mean age: 9.9 ± 11.9 [range: 1–48] months) were included in the study. Forty-three (75.4%) patients were in the first year and 56 (98.2%) patients were ≤ 3 years old. The demographic information is presented in Table 1.

**Table 1:** Demographic data and clinical features of the study participants

Variables	Total number of patients
Subjects, *n*	57
Sex (M/F), *n*	34/23
Age (mean ± SD), m	9.9 ± 11.9
Range, m	(1–48)
Facial PWS (%), *n*	
Bilateral side	16 (28.1%)
Right side	20 (35.1%)
Left side	21 (36.8%)
Glaucoma occurrence (%), *n*	
Bilateral eyes	4 (7.0%)
Right eye	29 (50.9%)
Left eye	24 (42.1%)
Bilateral PWS and glaucoma, *n*	4
Bilateral PWS and unilateral glaucoma, *n*	12
Mongolian spots, *n*	8 (14.0%)
Epilepsy, *n*	10 (17.5%)

### Systemic characteristics

**Fig. 1:**
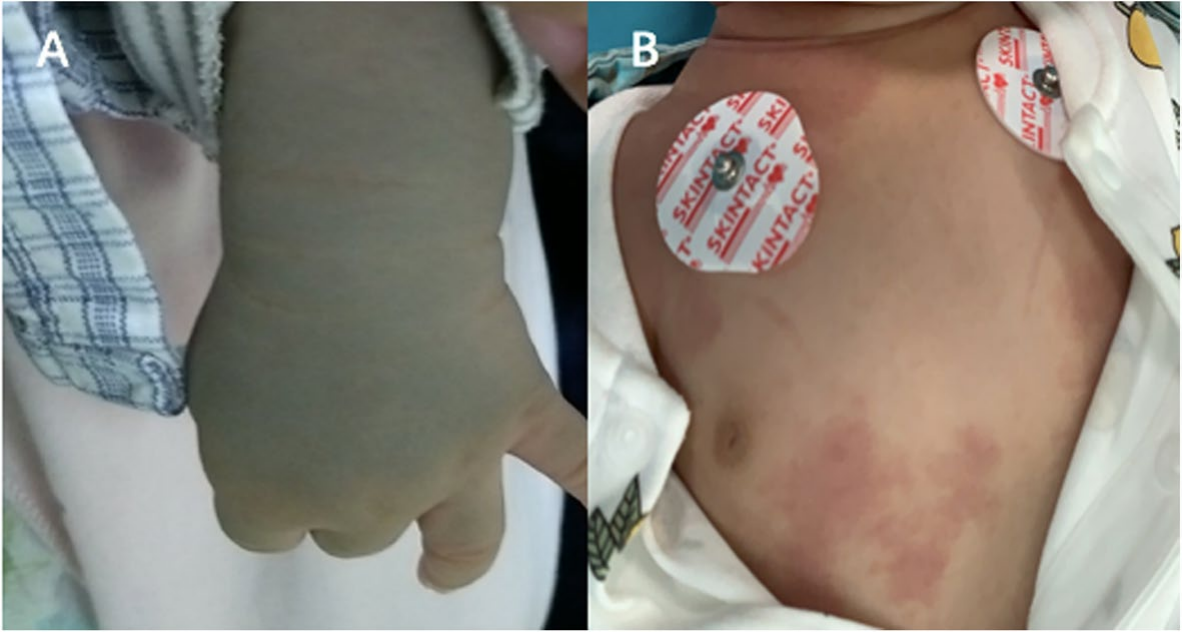
The skin changes of patients with SWS. Mongolian spots on the left forearm (**A**); PWS appeared in the neck and belly (**B**)

Twenty, 21, and 16 patients had right-sided, left-sided, and bilateral PWS, respectively. Of the 41 patients with unilateral facial PWS, glaucoma occurred on the same side of PWS. Of the patients with bilateral facial PWS, only four were diagnosed with bilateral glaucoma. Two patients had almost symmetrical PWS on both sides and both eyes were diagnosed with glaucoma. The other two patients with asymmetrical PWS, one side involved almost the entire trigeminal nerve area, and the other side involved the upper eyelid and part of the face. Eight patients (14.0%) had Mongolian spots on their bodies (Fig. 1A). These spots occurred in the back, buttocks, abdomen, feet, and limbs. Two patients, in addition to the facial PWS, also showed PWS on their scalp, neck, abdomen (Fig. 1B), feet, and buttocks. Ten patients (17.5%) had epilepsy.

### Ocular manifestations

Ocular manifestations were recorded in the conjunctiva, cornea, anterior chamber angle, pupil, lens, retina, and choroid (Table 2).

**Table 2:** Ocular manifestations of the study patients

Variables	The number of eyes
Glaucoma, *n*	61
Corneal changes (%), *n*	
Corneal oedema	36 (59.0%)
Corneal opacity	15 (24.6%)
Corneal haab line	13 (21.3%)
Conjunctival/episcleral haemangiomas (patients), *n*	30 (25)
Choroidal haemangiomas (patients), *n*	36 (33)
Iris insertion or hyperpigmentation in angle (patients), *n*	13 (10)
Trichiasis, *n*	6
Strabismus, *n*	3
Cataract, *n*	1
Pupil deformation, *n*	1
Tortuous expansion of retinal vessels, *n*	1

#### Ocular changes associated with glaucoma

**Fig. 2:**
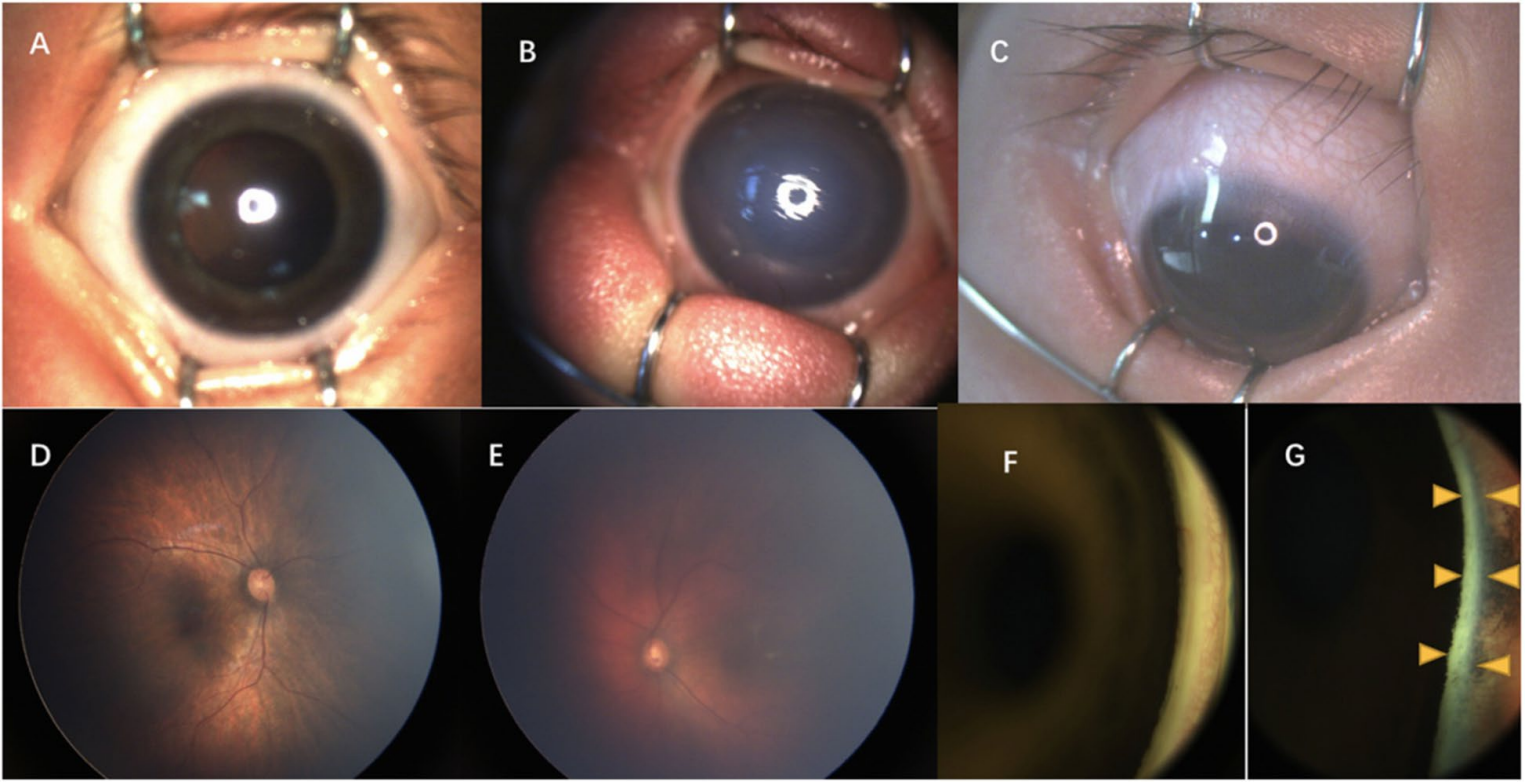
The ocular changes to the cornea, conjunctiva, fundus, and anterior chamber angle. **A** and **B** show the transparent cornea in the normal eye and the oedematous cornea in the eye with glaucoma. **C** shows the conjunctival/episcleral haemangiomas. **D** and **E** show the normal fundus and “tomato ketchup” fundus with choroidal haemangiomas. F and G are the anterior chamber angles examined by retcam, wherein **F** shows normal pigmentation and **G** shows hyperpigmentation (yellow arrow)

Among the 57 infants with SWS, 61 eyes were diagnosed with glaucoma and 53 eyes were normal. More than half of the eyes with glaucoma showed corneal oedema (Fig. 2A: the transparent cornea; Fig. 2B the oedematous cornea) (*n* = 36 eyes, 59.0%). Corneal opacity (*n* = 15 eyes, 24.6%) and corneal Haab lines (*n* = 13 eyes, 21.3%) were also commonly observed corneal changes. The eyes with glaucoma had higher IOP and greater corneal thickness, corneal diameter, axial length, and C/D (cup/disk) ratio (Table 3). The mean corneal diameter and thickness of the affected eyes was significantly larger than that of the unaffected eyes (12.2 ± 0.7 mm vs 10.8 ± 0.6 mm, *P* < 0.001; 681.2 ± 106.4 µm vs 578.2 ± 58.2 µm, *P* < 0.001). The affected eyes had higher IOP, larger axial length, and higher C/D ratio than the unaffected eyes (19.3 ± 6.2 mmHg vs 11.6 ± 4.2 mmHg, *P* < 0.001; 21.23 ± 1.93 mm vs 19.68 ± 1.61 mm, *P* < 0.001; and 0.57 ± 0.18 vs 0.24 ± 0.15, *P* < 0.001). Among the eyes affected by glaucoma, 21 eyes had IOP > 21 mmHg; the highest IOP recorded was 35 mmHg. Further, four patients had the axial length of > 24 mm in the eyes with glaucoma. The highest recorded axial length was 28.34 mm in a 21-month-old patient, and the affected eye showed -10 dioptre (D); the axial length of the contralateral eye was 21.97 mm and showed + 1.5 D.

**Table 3:** Comparison between eyes with glaucoma and unaffected eyes

Variables	Glaucomatous eyes	Unaffected eyes	*P* value
Total number, *n*	61	53	
IOP, mmHg	19.3 ± 6.2	11.6 ± 4.2	0.000
Corneal diameter, mm	12.2 ± 0.7	10.8 ± 0.6	0.000
Corneal thickness, µm	681.2 ± 106.4	578.2 ± 58.2	0.000
Axial length, mm	21.23 ± 1.93	19.68 ± 1.61	0.000
C/D ratio	0.57 ± 0.18	0.24 ± 0.15	0.000

#### Conjunctival/episcleral haemangioma, DCH and hyperpigmentation in the angle

**Fig. 3:**
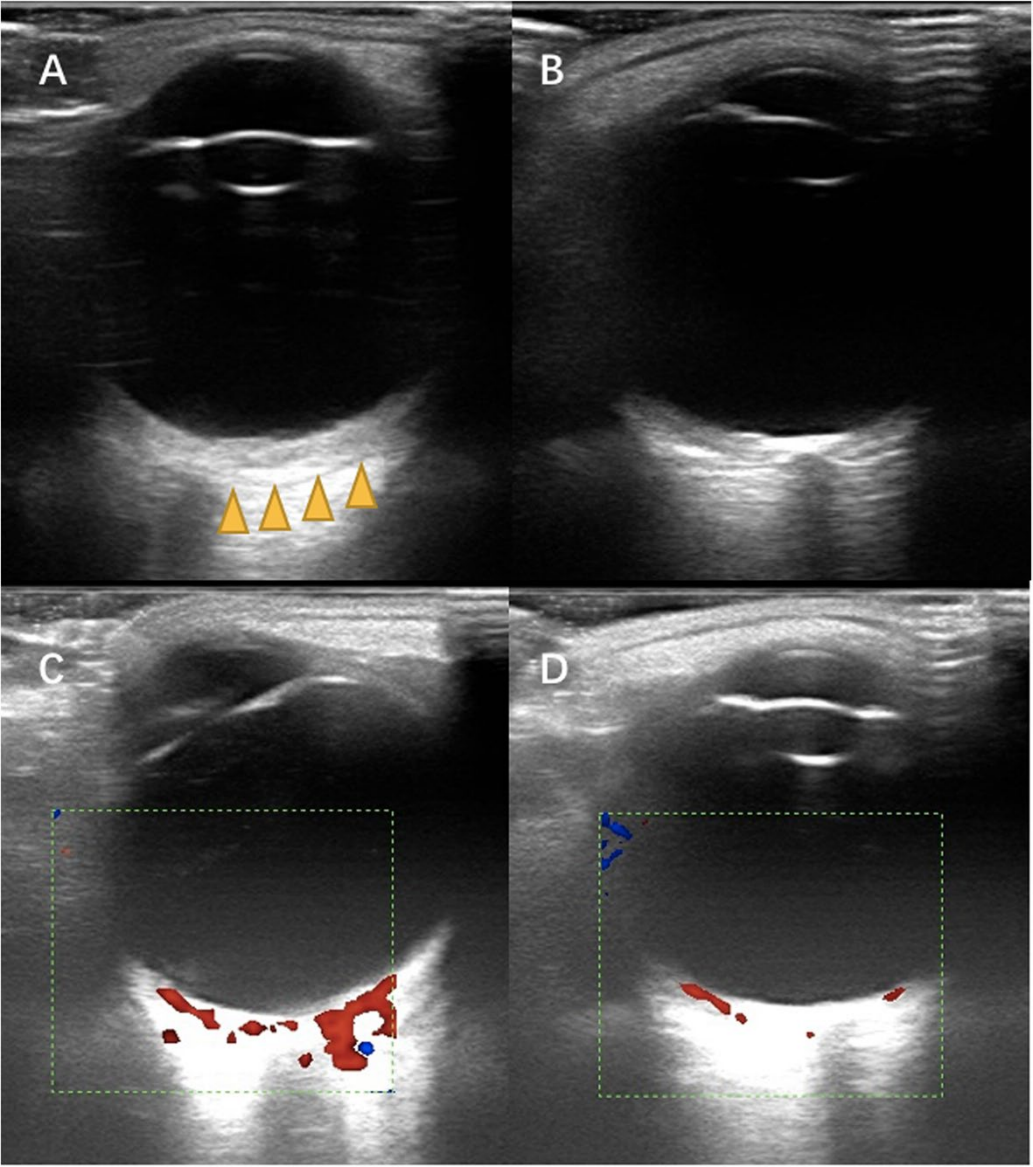
The ocular B-scan ultrasonography showed diffuse choroidal haemangiomas. Echo-enhanced thicker choroid with rich blood-flow signal in **A** (the yellow arrow) and **C**. The normal choroidal thickness and blood-flow signal in **B** and **D**

Conjunctival/episcleral haemangioma (Fig. 2C) occurred in 30 eyes of 25 patients. Twenty patients had unilateral conjunctival/episcleral haemangioma and ipsilateral glaucoma. Five patients had bilateral conjunctival/episcleral haemangioma. Of these five patients, one was diagnosed with bilateral glaucoma and bilateral PWS; two had bilateral PWS, but unilateral glaucoma; and two patients had unilateral PWS and glaucoma, but bilateral conjunctival/episcleral haemangioma. DCH occurred in 36 eyes of 33 patients. Thirty patients had unilateral DCH and ipsilateral glaucoma. Three patients had bilateral DCH. Of these three patients, two had bilateral PWS and glaucoma, while one had bilateral PWS and unilateral glaucoma. In patients with DCH, fundus examination showed dark-red diffused localized areas resembling ‘tomato ketchup’ in 26 eyes (Fig. 2D: the normal fundus; Fig. 2E the “tomato ketchup” fundus). Ocular B ultrasound examination showed an echo-enhanced thicker choroid with rich blood-flow signal in the eyes with DCH (Fig. 3).

Thirteen eyes (10 patients, 17.5%) showed anterior insertion of the iris or hyperpigmentation in the anterior chamber angles (Fig. 2F: the anterior chamber angles with normal pigmentation; Fig. 2G: the anterior chamber angles with hyperpigmentation). All cases occurred in eyes with glaucoma. Among the eight patients with Mongolian spots, six patients (75.0%) got iris anterior insertion or hyperpigmentation in the anterior chamber angles.

#### Others

Six (9.8%) patients had trichiasis, and all of them had unilateral glaucoma. The minimum age of patients was 8 months. Five of them had trichiasis in the ipsilateral side with glaucoma, one patient had unilateral glaucoma and bilateral trichiasis. Three patients had strabismus and all were cases of exotropia. In a 2-month-old boy who was diagnosed with bilateral glaucoma and PWS, the left eye showed pupil deformation and lens opacity. Another 12-month-old boy showed tortuous expansion of the retinal vessels in the eye with glaucoma.

## Discussion

SWS is an ocular and neurocutaneous disorder. All SWS patients in the study had PWS on the face and secondary glaucoma. Among them, 17.5% and 14.0% patients had neurological symptoms and Mongolian spots, respectively. Other ocular manifestations included DCH, conjunctival/episcleral haemangiomas, iris anterior insertion or hyperpigmentation in anterior chamber angle, and glaucoma-related ocular changes such as corneal oedema, larger corneal diameter, longer axial length, and higher C/D rate. The sex distribution showed a slight male preponderance (59.6%), which is close to the reported sex distribution (63.4%) by Sharan et al. [11]. However, in one report by Bayoumi et al., the proportion of male patients was 45% [12]. The mean age of patients was 10 months, which is similar to the age of patients with primary congenital glaucoma [13, 14], and the average age is also within one year.

Glaucoma is a common ocular complication of patients with SWS; it causes considerable harm to vision and hence needs significant attention. One study reported that 48% patients with SWS have secondary glaucoma [15]. It has been reported that the incidence of glaucoma caused by SWS follows a bimodal distribution. About 60% patients’ glaucoma is onset in infancy and is often accompanied by enlargement of the eyeball and corneal diameter. About 40% patients’ glaucoma is onset in childhood or adulthood, without corneal and eyeball enlargement [15, 16]. The main cause of the disease in infants is the abnormal angle caused by the abnormal development of the mesoderm, while the cause of the latter is an increase of the scleral venous pressure [17]. In this study, glaucoma occurred mainly in infants aged ≤ 4 years. Among them, corneal oedema occurred in > 50% patients, and the corneal diameter, axial length, and C/D rate were significantly larger than that of normal eyes. These findings were similar to those of primary congenital glaucoma [13, 14]. This is because, in infancy, the plasticity of the eyeball collagen fibres is significant. When the IOP increases continuously, secondary ocular changes occur, including globe and cornea enlargement, Descemet’s membrane breakage, and corneal oedema. Bayoumi et al. [12] reported 22 patients with PWS and 44 eyes were in their study. Among them, 34% and 36% of the study eyes were diagnosed with glaucoma and no glaucoma, respectively, and the eyes with glaucoma had higher IOP and larger corneal diameter, axial length, and higher C/D ratio than those without glaucoma, which is in accordance with our study. For these patients, topical anti-glaucoma drugs are not adequate for good IOP control, and surgical treatments are mostly needed. Goniotomy and trabeculotomy are the most commonly used surgical methods [18].

Though the IOP of eyes with glaucoma was significantly higher than that of unaffected eyes in this study, only 34.4% eyes had an IOP > 21 mmHg. The IOP of healthy neonates is lower than the average adult IOP value [19] and reaches the adult level by adolescence [20]. The measurement of IOP in infants is difficult to operate and fluctuates greatly. It is greatly affected by the cooperation of children, eye movement, anaesthesia, eyelid opener, and corneal condition. Thus, the diagnosis of infantile glaucoma cannot be dependent on IOP alone. It should be diagnosed according to the overall clinical manifestations and examination results [10].

DCH is another frequent ocular manifestation in SWS patients. It arises from the choroid and is a benign vascular tumour. Some reports showed that it occurred in 20–70% patients with SWS, which is the commonest posterior segment abnormality [21]. In our study, 33 (57.9%) patients had DCH. The fundus appeared as has been reported dark-red diffused localised areas resembling ‘tomato ketchup’ [22]. DCH usually present asymptomatically and can be found by ophthalmoscopy examination or B-scan ultrasound examination. Enhanced depth imaging optical coherence tomography (EDI-OCT) can show the choroid in vivo, and it can be seen that choroidal thickening occurs obviously in the eyes with DCH [23, 24]. Fluorescein angiography (FFA) and indocyanine green angiography (ICGA) can present DCH better [25], but both are invasive and the cooperation of young patients can be challenging.

In addition to glaucoma and DCH, we also found other ocular changes including conjunctival/episcleral haemangiomas, trichiasis, strabismus, cataract, tortuous expansion of retinal vessels, and pupil deformation. Twenty-five (43.9%) patients had conjunctival/episcleral haemangiomas in the study. Javaid et al. [18] reported that episcleral vessel dilatation could be observed in 50% patients with SWS, which was similar to our study. Emelen et al. [26] summarised the clinical findings of 19 patients with SWS and found that 10 patients had conjunctival/episcleral haemangioma, one had iris heterochromia, one had retinal detachment, and four had strabismus. Besides, Ali et al. also reported that a patient with SWS had cataract [8].

PWS is a typical skin change that occurs in the distribution of the ophthalmic branch of the trigeminal nerve in patients with SWS. In this study, the facial vessels on both sides had similar probability. We found the PWS also appeared on the scalp, neck, feet, and buttocks. Bayoumi et al. reported that five patients with PWS showed involvement of the trunk and extremities [12]. Eight patients were diagnosed with phakomatosis pigmentovascularis (PPV) and SWS with the characteristics of Mongolian spots, PWS, and glaucoma. Six of them showed iris anterior insertion or hyperpigmentation in the anterior chamber angles. PPV is a rare congenital condition with the combination of capillary abnormalities and dermal melanocytosis. It is classified into three subtypes as cesioflammea, spilorosea, and cesiomarmorata. Cesioflammea presents with nevus flammeus and Mongolian spots and may be accompanied by glaucoma. The mechanism of glaucoma in PPV are the immature angle and Schlemm canal structure, the anterior insertion of the iris and ciliary body, pigmentary alterations of the trabecular meshwork [27]. The neurological complications of SWS include epilepsy, migraine, stroke-like episodes, and learning and behavioural difficulties [28]. Seizures are the main manifestations of patients with central nervous system involvement, and most seizures occur in the first 2 years of life [26]. A study found that 75% seizures presented in the first year of their life, 11% during the second year, and only 14% after the second year [29].

Our study has some limitations. First, the relatively small number of patients limits drawing robust and generalizable conclusions. Second, the age range is narrow and cannot reflect the situation of patients of a different age group. Third, patients were too young to undergo gonioscopy examination, and the angle results by retcam examination were not accurate enough. Last, we did not get the cranial magnetic resonance imaging results of all patients, so we were unable to summarise and explain the intracranial lesions.

## Conclusions

In the infants with PWS and glaucoma secondary to SWS, monocular glaucoma is more common and on the same side with PWS. About half of the patients show DCH, and conjunctival/episcleral haemangiomas. Glaucomatous eyes have higher IOP and C/D ratio, larger corneal diameter and axial length and thicker cornea. Patients with Mongolian spots have higher incidence of iris anterior insertion or hyperpigmentation in anterior chamber angle.

## Data Availability

The datasets generated and/or analysed during the current study are not publicly available due to the fact that the following-up research including treatment and effect on these patients is still going on, but are available from the corresponding author on reasonable request.

## Abbreviations

SWS: Sturge–Weber Syndrome
PWS: Port-wine stain
IOP: Intraocular pressure
C/D: Cup/disk
DCH: Diffuse choroidal haemangiomas
EDI-OCT: Enhanced depth imaging optical coherence tomography
FFA: Fluorescein angiography
ICGA: Indocyanine green angiography
